# Engraftment of Human Pluripotent Stem Cell-derived Progenitors in the Inner Ear of Prenatal Mice

**DOI:** 10.1038/s41598-018-20277-5

**Published:** 2018-01-31

**Authors:** Hiroki Takeda, Makoto Hosoya, Masato Fujioka, Chika Saegusa, Tsubasa Saeki, Toru Miwa, Hideyuki Okano, Ryosei Minoda

**Affiliations:** 1 Departments of Otolaryngology-Head and Neck Surgery, Kumamoto University Graduate School of Medicine, 1-1-1 Honjo, Chuoku, Kumamoto city, Japan; 2 Departments of Otolaryngology-Head and Neck Surgery, Keio University School of Medicine, 35 Shinanomachi, Shinjuku-ku, Tokyo, Japan; 3 Department of Physiology, School of Medicine, Keio University, 35 Shinanomachi, Shinjuku, Tokyo, Japan; 4 Departments of Otolaryngology-Head and Neck Surgery, Middle Ear and Inner Ear Surgical Center, JCHO Kumamoto General Hospital, 10-10 Tori Machi, Yatsushiro, Kumamoto, 866-8660 Japan

## Abstract

There is, at present, no curative treatment for genetic hearing loss. We have previously reported that transuterine gene transfer of wild type CONNEXIN30 (CX30) genes into otocysts in CX30-deleted mice could restore hearing. Cell transplantation therapy might be another therapeutic option, although it is still unknown whether stem cell-derived progenitor cells could migrate into mouse otocysts. Here, we show successful cell transplantation of progenitors of outer sulcus cell-like cells derived from human-derived induced pluripotent stem cells into mouse otocysts on embryonic day 11.5. The delivered cells engrafted more frequently in the non-sensory region in the inner ear of CX30-deleted mice than in wild type mice and survived for up to 1 week after transplantation. Some of the engrafted cells expressed CX30 proteins in the non-sensory region. This is the first report that demonstrates successful engraftment of exogenous cells in prenatal developing otocysts in mice. Future studies using this mouse otocystic injection model *in vivo* will provide further clues for developing treatment modalities for congenital hearing loss in humans.

## Introduction

A genetic defect is the most common cause of hearing loss at birth and in childhood. These hearing losses have a profound negative impact on daily living. Numerous causative genes for genetic hearing loss have been identified. However, at present, there are no truly curative therapies for this condition. When considering curative treatments for genetic hearing loss, gene- and cell-based therapies might be good options, and there have been several recent reports on successful treatment in mice using embryonic gene therapy, neonatal gene therapy, and neonatal antisense oligonucleotide therapy^1,2^. However, there are only very few reports describing cell-based therapies for genetic hearing loss.

CONNEXINs (CXs) are gap junction proteins that play a crucial role in hearing, and mutations in CXs-encoding genes are responsible for over 50% of cases of hereditary hearing loss in humans^3^. CXs function as intracellular communicators in transporting cAMP, nucleotides, calcium ions, inositol triphosphate, and small molecules for cellular homeostasis^4^. In the mammalian cochlea, the CX26 and CX30 are expressed in the non-sensory epithelium; the supporting cells, stria vascularis, spiral ligament, spiral limbus, and these CXs are co-assembled to form homotypic and heterotypic/heteromeric gap junctions^5,6^. A mutation in the *GJB2* gene, which encodes CX26^7–9^, and a mutation in the *GJB6* gene, which encodes CX30^9,10^, are major common genetic causes of nonsyndromic sensorineural hearing loss in humans. The deficiencies of either CX26 or CX30 in mice can cause congenital deafness with cochlear developmental disorders, hair cell degeneration, and the reduction of the endocochlear potential (EP)^11,12^.

Regarding treatment for CX-related genetic hearing loss, several successful *in vivo* gene therapy treatments have been reported^2,13^. While cell transplantation therapy might also be an option for treatment of genetic hearing loss, no previous reports have described the use of *in vivo* cell transplantation therapy for genetic hearing loss. However, a few reports have described successful differentiation of stem cells *in vitro* into cells expressing CX26 or CX30. Fukunaga *et al*. reported on the successful generation of CX26- and CX30-expressing cells from mouse-derived induced pluripotent stem cells (miPSC)^14^. More recently, Hosoya *et al*. reported on the successful generation of outer sulcus cell-like cells (OSCs), which express PENDRIN, CX30, CX26, and other outer sulcus cell markers, from human-derived iPS cells (hiPSCs)^15^.

In this study, we thus set out to explore the feasibility of cell transplantation therapy for CXs-related hearing loss. To that end, we first performed the delivery of progenitors of OSCs into the otocysts of wild type and CX30-deleted mice, and, subsequently, examined the engraftment of the delivered cells into the inner ears.

## Results

### *In vitro* cell culture

**Figure 1:**
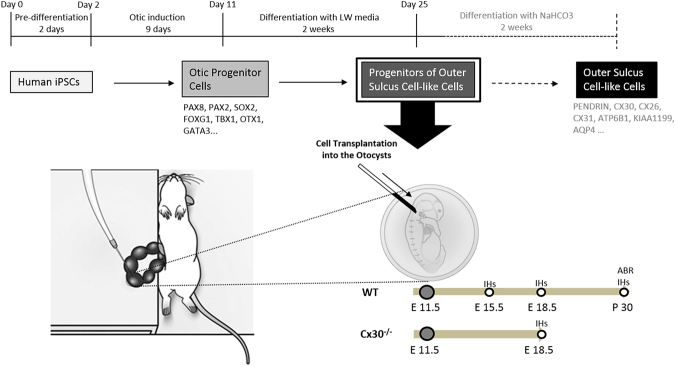
The upper schema illustrates a cell culture of hiPSCs *in vitro* and cell transplantation into the otocysts *in vivo*. The hiPSCs were cultured for 25 days and differentiated into progenitors of OSCs. We utilized these progenitors of OSCs as donor cells. To confirm the developmental potency of these cells, they were cultured for another 2 weeks. The lower schema illustrates the otocystic injection procedure, and the schedule of the *in vivo* experiment. IHC: immunohistochemical analysis; ABR: auditory brain stem response.

**Figure 2:**
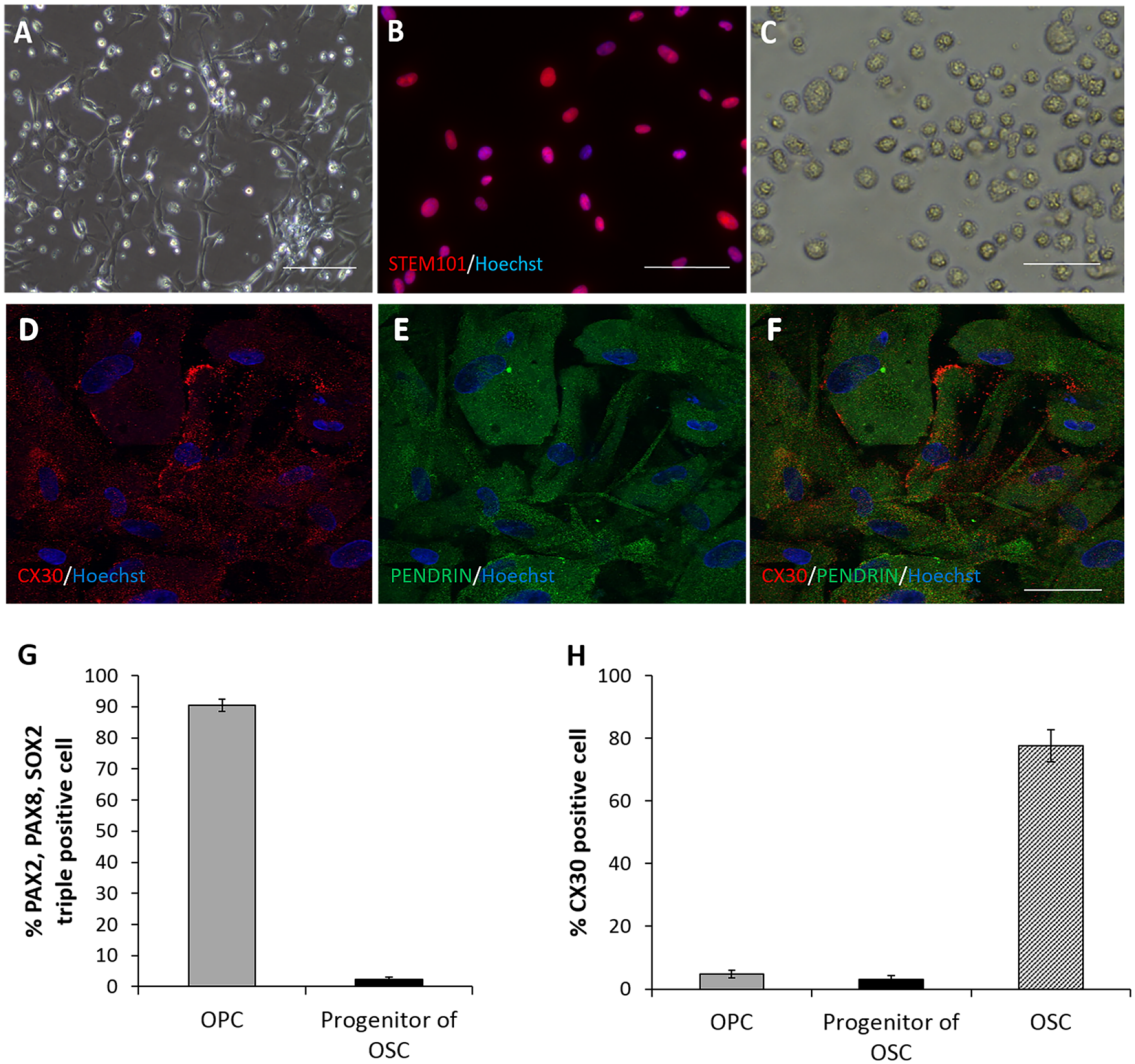
(**A**) The image shows adhesive progenitors of OSCs in culture. The bar indicates 50 μm. (**B**) The image shows the reactivity of progenitors of OSCs with STEM101 in the nuclei. STEM101 (red) and Hoechst (blue) are co-expressed in all cells. The bar indicates 50 μm. (**C**) The image shows progenitors of OSCs dissociated into single cells with trypsin. The size of the cells is 10–25 μm. The bar indicates 50 μm. (**D**) Image of OSCs immunostained with CX30 (red). Nuclei were counterstained with Hoechst (blue). (**E**) Image of OSCs immunostained with PENDRIN (green). Nuclei were counterstained with Hoechst (blue). (**F**) Merged image of (**D**) and (**E**). Red: CX30; Green: PENDRIN; Blue: nuclei. Scale bars indicate 50 μm. (**G**) The graph shows percentages of cells positive for otic markers (PAX2, PAX8, and SOX2) among OPCs and progenitors of OSCs *in vitro*. (**H**) The graph shows percentages of CX30-positive cells in OPCs, progenitors of OSCs, and OSCs *in vitro*.

Differentiation (otic induction) of hiPSCs was initiated on day 2 and finished on day 11 and was achieved with FGF2, FGF3, FGF10, FGF19, and BMB4. The induced otic progenitor cells (OPCs) expressed PAX8, PAX2, SOX2, FOXG1, TBX1, OTX1, and GATA3, as confirmed by immunocytochemical analysis and RT-PCR^15^ (Fig. 1). Then, the OPCs were differentiated into progenitors of outer sulcus cell-like cells (OSCs), which were used for transplantation. As observed through immunohistochemical analysis, 90.46 ± 2.04% of OPCs expressed PAX2, PAX8, and SOX2, while approximately 2.24 ± 0.82% of the progenitors of OSCs expressed these markers *in vitro* (Fig. 2G). The progenitors of OSCs were positive for human-nuclei specific antibody (STEM101) *in vitro* (Fig. 2A–C). The progenitors of OSCs were then differentiated to OSCs with weekly NaHCO3 for 2 weeks. The induced OSCs expressed PENDRIN, CX30 (Fig. 2), CX26, CX31, ATP6B1, KIAA1199, AQP4, and other outer sulcus cell markers^15^ (Fig. 1). As observed through immunohistochemical analysis, 4.80 ± 1.19% of OPCs, 3.09 ± 1.23% of progenitors of OSCs, and 77.58 ± 5.13% of OSCs expressed CX30 *in vitro* (Fig. 2H).

### Cell transplantation to WT mice

**Figure 3:**
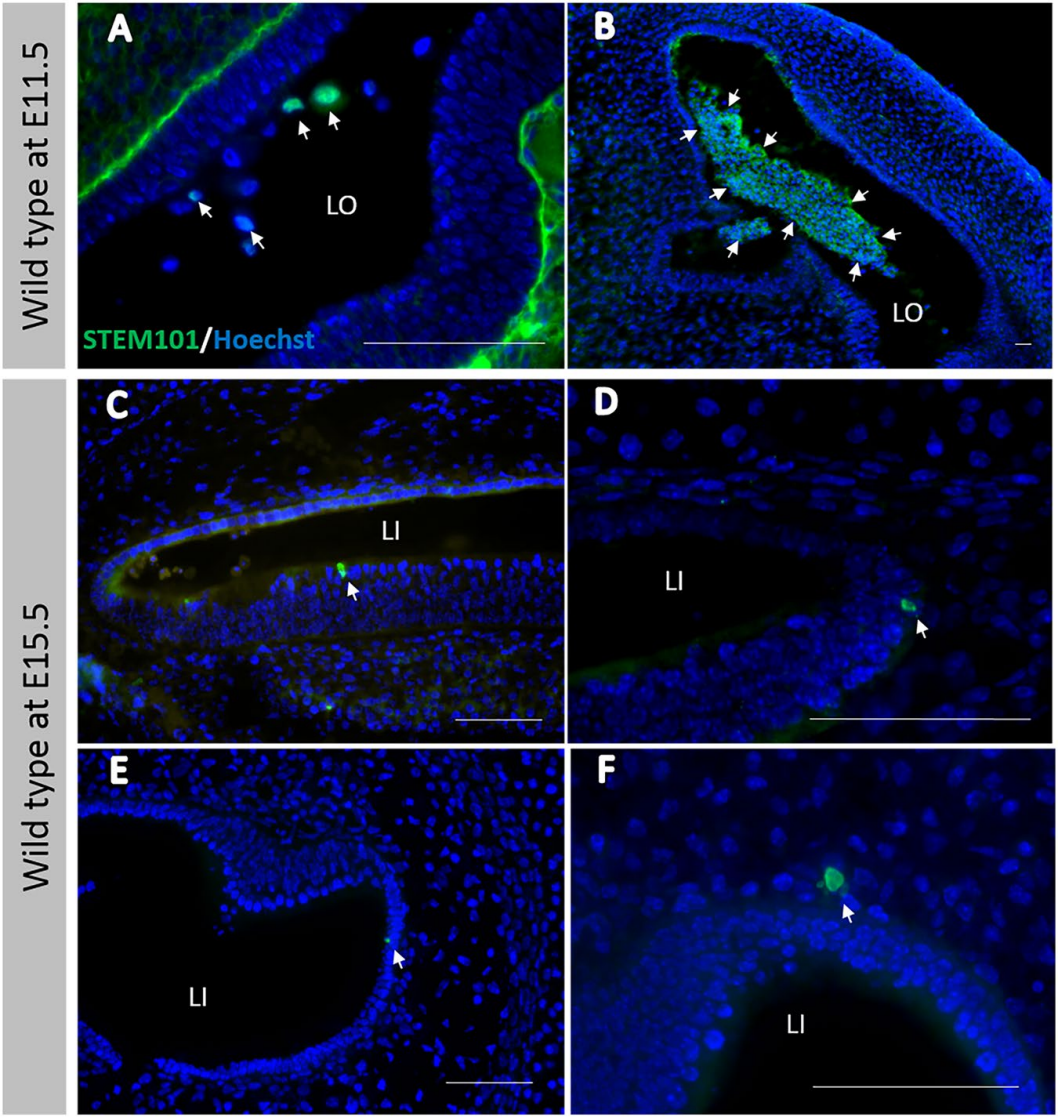
(**A**) STEM101-positive cells in the lumen of the treated inner ears immediately after cell delivery into the otocyst. (**B**) Agglomerated STEM101-positive cells in the lumen of the treated inner ear (arrows) immediately (approximately 1 h) after cell delivery. (**C**) A STEM101-positive cell found in the cochlear epithelium (arrow) at the middle turn. (**D**) A STEM101-positive cell found in the cochlear lateral wall (arrow) at the apical turn. (**E**) A STEM101-positive cell in the semicircular canal epithelium (arrow). (**F**) A STEM101-positive cell in the subepithelium (arrow) at the treated semicircular canal. LO: Lumen of the otocyst. LI: Lumen of the inner ear. Green: STEM101; Blue: Hoechst. Scale bars indicate 100 μm.

**Figure 4:**
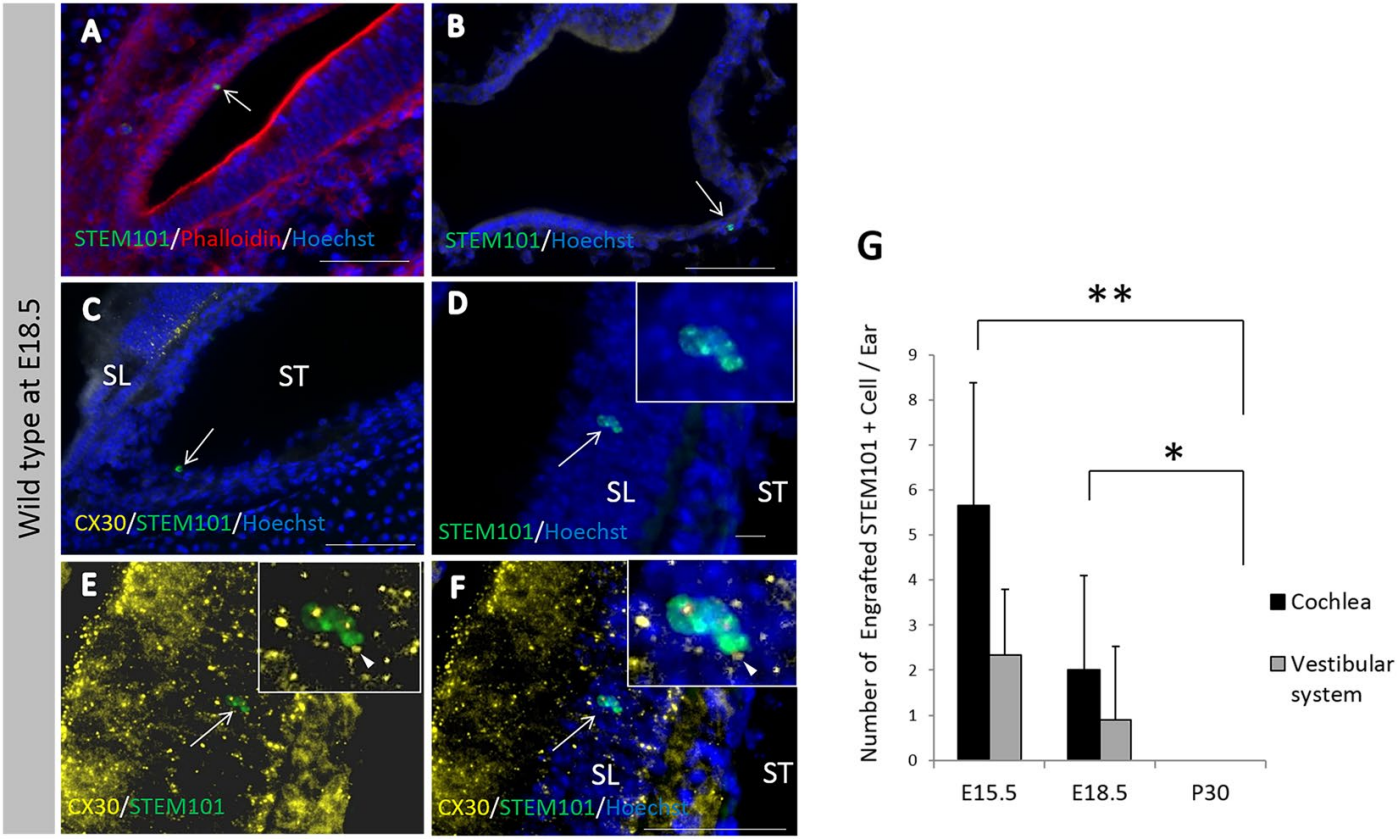
(**A**) A STEM101-positive cell in the LW of the cochlea (arrow) at the apical turn. (**B**) A STEM101-positive cell in the vestibule (arrow). (**C**) A STEM101-positive cell that was negative for CX30 staining found in the ST of the middle turn of the cochlea (arrow). (**D–F**) A STEM101-positive cell (arrow) in the SL of the cochlear apical turn. The arrowhead indicates CX30 staining around the nucleus. (**G**) The graph shows the numbers of STEM101-positive cells in the treated inner ears in the WT-treated group at E15.5, E18.5 and P30. *n* = 10 for E18.5 and *n* = 5 for both E15.5 and P30. LW: lateral wall; SL: spiral limbus; ST: scala tympani; Green: STEM101; Red: Phalloidin; Yellow: CX30; Blue: Hoechst. Scale bars indicate 100 μm. *P < 0.05; **P < 0.01.

After transplantation into WT mice otocysts, all engrafted cells, which were defined as cells that were double-positive for both STEM101 and nucleic staining, were only detectable in the lumen of the treated inner ears immediately after cell injections (Fig. 3A,B) or in the non-sensory region in the inner ears on E15.5 and E18.5 in treated WT mice. Further, on E15.5, STEM101-positive cells were detectable in the cochlear non-sensory epithelium (Fig. 3C,D), in the non-sensory epithelium associated with the semicircular canal (Fig. 3E), and in the semicircular canal non-sensory subepithelium (Fig. 3F). On E18.5, STEM101-positive cells were detectable in the non-sensory region in the cochleae and in the vestibular system; the engrafted cells were detectable in the lateral wall (Fig. 4A), the spiral limbus (Fig. 4D–F), the scala vestibuli, the lining cells of the scala tympani (Fig. 4C), Rosenthal’s canal, the modiolus, the vestibule (Fig. 4B), and the semicircular canals. CX30 protein expression in STEM101-positive cells was assessed in three of the 10 E18.5 embryos in the WT-treated group. In one embryo we observed a STEM101-positive cell in the spiral limbus (Fig. 4D–F) that was positive for CX30 staining, while the other engrafted STEM101-positive cells were all negative for CX30 staining (Fig. 4C) in the embryos we assessed. At P30, STEM101-positive cells were not detectable at all in the treated inner ears (Supplementary Figure 2A). For quantification, we counted the number of engrafted cells in the inner ear after transplantation. On E15.5, we found from 2 to 16 cells per mouse engrafted in the treated inner ear; we found that 2.33 ± 1.33 and 0.67 ± 0.67 cells per mouse were engrafted in the treated cochlea and vestibule, respectively. The number of engrafted cells observed in the inner ear was highest on E15.5. This number decreased on E18.5, which showed 0 to 10 cells per mouse (2.00 ± 2.10 engrafted in the cochlea epithelium). In WT mice, 80% (8/10) had engrafted cells at E18.5 after treatment. No engrafted cells were detected in the treated inner ears on P30 (Fig. 4G, Table 1). ABR testing revealed that there was no significant difference in auditory thresholds between treated sides and non-treated sides on P30 (Supplementary Figure 2B).

**Table 1:** The Numbers of Engrafted Cells in the Inner Ears.

Stage of Assessment	E15.5				E18.5		P30
Location	Cochlea		Vestibular System		Cochlea	Vestibular System	Inner Ear
	Epithelium	Subepithelium	Epithelium	Subepithelium			
WT	2.33 ± 1.33 (n = 5)	3.33 ± 1.45 (n = 5)	0.67 ± 0.67 (n = 5)	1.67 ± 0.88 (n = 5)	2.00 ± 2.10 (n = 10) *CX30 ( + ) Cell; at Spiral Limbus (n = 1/3)	0.90 ± 1.22 (n = 10)	0 (n = 5)
CX30^−/−^	NP	NP	NP	NP	5.60 ± 1.62 (n = 5) *CX30 ( + ) Cells; at Spiral Limbus or Reissner’s Membrane (n = 2/2)	1.00 ± 1.10 (n = 5)	NP

Data indicate the mean ± SE of the engrafted cells with STEM101 positive. The asterisks indicate the locations where CX30 (+) cells were detectable, and the numbers in the parentheses indicate the numbers of animals which had CX30-positive cells/the number of animals on which we performed immunostaining with CX30. “n” indicates the number of animals on which we performed immunostaining with STEM101. “NP” stands for not performed.

### Transplantation to Cx30^−/−^ mice

**Figure 5:**
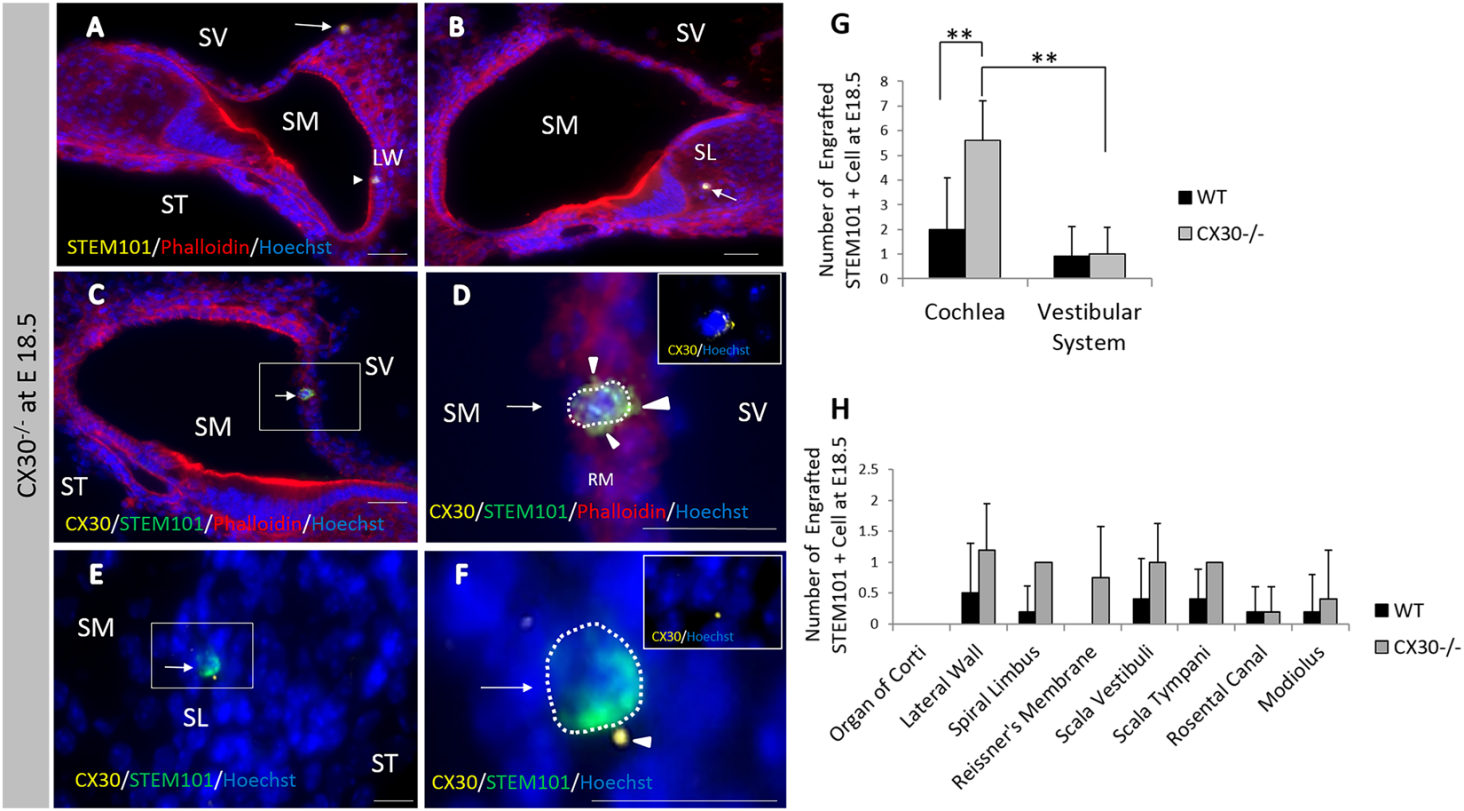
(**A**) STEM101-positive cells found in the LW (arrowhead) and in the lining cells of the SV at the cochlear apical turn (arrow). Red: Phalloidin; Yellow: STEM101; Blue: Hoechst. (**B**) A STEM101-positive cell in the SL epithelium in the cochlear basal turn (arrow). Red: Phalloidin; Yellow: STEM101; Blue: Hoechst. (**C**) A STEM101-positive cell (arrow) in the RM in the cochlear middle turn. Green: STEM101; Red: Phalloidin; Yellow: CX30; Blue: Hoechst. (**D**) Higher magnification of the square area in (**C**). The STEM101-positive cell (arrow) expresses CX30 protein (arrowheads) around the nuclei (white dotted line) in the cytoplasm. (**E**) A STEM101-positive cell (arrow) in the SL in the cochlear basal turn. Green: STEM101; Red: Phalloidin; Yellow: CX30; Blue: Hoechst. (**F**) Higher magnification of the square area in (**E**). The STEM101-positive cell (arrow) expresses CX30 protein (arrowheads) around the nuclei (white dotted line) in the cytoplasm. LW: lateral wall; RM: Reissner’s membrane; SL: spiral limbus; SM: scala media; ST: scala tympani. Scale bars indicate 100 μm. (**G**) The graph shows the number of STEM101-positive cells in the treated cochlea and the vestibular system in the WT-treated and CX30^−/−^ treated groups on E18.5. The numbers of STEM101-positive cells in the CX30^−/−^ mouse cochleae were significantly higher than those in the vestibular system, and the numbers of STEM101-positive cells in in the CX30^−/−^ mouse cochleae were significantly higher than those in WT mouse cochleae. *n* = 10 for the WT-treated group, *n* = 5 for the CX30^−/−^ treated group. **P < 0.01. (**H**) The graph shows the number of engrafted STEM101-positive cells in the treated cochlea in the WT-treated and CX30^−/−^ treated groups on E18.5. *n* = 10 for the WT-treated group, *n* = 5 for the CX30^−/−^ treated group.

On E18.5, STEM101-positive cells were detectable in the non-sensory region in the cochleae (Fig. 5A–F) and in the vestibular system (Fig. 5G), but not in the sensory regions or in the lumen of the treated inner ears. These positive cells were located in the lateral wall (Fig. 5A), the spiral limbus (Fig. 5B), Reissner’s membrane (Fig. 5C,D), the scala vestibule (Fig. 5A), the scala tympani, Rosenthal’s canal, the modiolus, the vestibule, and the semicircular canals. CX30 protein expression in STEM101-positive cells was assessed in two out of the five E18.5 embryos in the CX30-treated group. In this assessment, CX30-positive cells were detectable in the spiral limbus and in Reissner’s membrane (one cell per embryo) (Fig. 5C–F, Table 1), while all other engrafted STEM101-positive cells were negative for CX30 staining. For quantification, we counted the number of engrafted cells in the inner ear. On E18.5, all of the Cx30^−/−^ embryos (n = 5) had engrafted cells at E18.5 after transplantation, showing 5–10 cells per mouse engrafted in the treated inner ear; specifically, 5.60 ± 1.62 and 1.00 ± 1.10 cells per mouse were engrafted in the treated cochlea and vestibule, respectively (Table 1). The number of STEM101-positive cells in the cochlea in the CX30^−/−^ treated group was significantly higher than that in the vestibular system in the CX30^−/−^ treated group on E18.5 (Fig. 5G). Moreover, the number of STEM101-positive cells in the cochlea in the CX30^−/−^ treated group was significantly higher than that in the cochlea in the WT-treated group on E18.5 (Fig. 5G).

## Discussion

We demonstrate here that hiPSCs-derived progenitors of OSCs were successfully transplanted into WT mouse otocysts or CX30^−/−^ mouse otocysts. This is the first report of successful cell transplantation into mouse otocysts.

The most suitable donor cells for cell transplantation *in vivo* for genetic hearing loss treatment may differ, depending on the causative genes of the hearing loss. When considering cell therapy for genetic hearing loss caused by CXs mutations, the suitable donor cells for cell transplantation therapy may be the cells that were destined to express CXs; donor cells may ideally be cells that are destined to differentiate into non-sensory cells in the inner ear, because sensory cells normally do not express CXs. Progenitors of OSCs are known to differentiate into OSCs, which express CX30, CX26, PENDRIN, among others, *in vitro*^15^. Therefore, we utilized the progenitors of OSCs as donor cells in this study. Differentiation levels of donor cells also may affect subsequent engraftment rates of donor cells into target organs. There are several reports describing the relation between differentiation stages of donor cells and engraftment rates of those cells. Ganat *et al*. transplanted dopaminergic neurons at three different developmental stages, which were derived from mouse embryonic stem cells (ESCs), into the striatum of hemiparkinsonian mice^16^. Consequently, the moderately differentiated ESC-derived DA neurons showed the greatest amount of DA neuron survival and robustly induced recovery of motor deficits. Kozubenko *et al*. transplanted undifferentiated human embryonic stem cells (hESCs) or partially differentiated hESC-derived neural precursors (NPs) into the injured rodent brain, caused by middle cerebral artery occlusion^17^. They reported that cell migration towards the lesion site was only detectable when cells with higher differentiation levels were transplanted, while the survival of the grafted cells was lower when cells with higher differentiation levels were transplanted. They also reported that tumor formation was only detectable when cells with lower differentiation levels were transplanted. Hildebrand *et al*. transplanted mouse ES cells that were undifferentiated or partially differentiated towards the neuroectoderm lineage into the guinea pig scala media^18^, and found that the rate of survival of the transplanted cells in the inner ears decreased with the differentiation level of the transplanted cells. These previously reported results suggest that cells at lower differentiation levels have a risk of tumor formation, and cells at higher differentiation levels have higher engraftment rates into the target sites, although those cells may have lower cell survival rates. Considering both the survival rate and the engraftment rate, moderately differentiated cells appear to be more preferable for cell transplantation *in vivo*. Therefore, we decided to utilize progenitors of OSCs^15^ for cell transplantation into the otocysts in this study.

The progenitors of OSCs, which were delivered into the otocysts *in vivo*, successfully engrafted both in wild type mice and CX30^−/−^ mice in this study. The number of engrafted cells in the cochlea in the CX30^−/−^ treated group on E18.5 was significantly higher than that in WT-treated mice in this study. The possible cause of this result is that CXs deficiency may affect the permeability of the lining cells of the lumen in the developing cochleae. Gap junctions constitute a critical pathway for intercellular communication^19–22^. CXs are the protein subunits that form gap junctions, and the most common CXs in the cochlea are CX26 and CX30, which coassemble in gap junction channels^20^. Interestingly, overexpression of CX26 decreased paracellular permeability in a monolayer model utilizing Caco-2 human colonic cells^23^. Considering this effect of CX26 on paracellular permeability, CX26 deficiency may increase the paracellular permeability and, as a consequence, the migration of the cells. Moreover, it has been shown previously that the CX30^−/−^ mice^12^, which we utilized in this study, have reduced CX26 expression^24,25^. These mice therefore probably have increased paracellular permeability, resulting in increased migration of the donor cells in the cochleae.

The number of engrafted STEM101-positive cells in the cochlea in the CX30^−/−^ treated group was significantly higher than that in the semicircular canals and the vestibule in the CX30^−/−^ treated group on E18.5, while there was no significant difference between these regions in the WT-treated group. There are two possible explanations for this difference in engrafted cell numbers between the cochlea and vestibular system. One possible explanation is that the effects of the CX30 deletion may be different in the cochlea compared with the vestibular system. Previous studies have shown that CX26 deletion in mice, which also had decreased or absent CX30 in the vestibular organ, did not cause hair cell damage in the vestibular organ postnatally^26^. In addition, Qu *et al*. reported that homozygous CX30-gene knockout mice^12^ showed a significant loss of cochlear and vestibular hair cells, but only in the saccule, while there was no significant loss of hair cells in the utricle or the ampulae^27^. Meanwhile, previous studies have shown that progenitor cells can engraft in damaged tissue more easily than in non-damaged tissue^28^. Taken together, the more significant damage in the cochlea compared with that in the vestibular system may cause more frequent cell engraftment in the cochlea. Another possible explanation may be related to the donor cells. We utilized progenitors of OSCs as donor cells, which are known to differentiate into OSCs in the cochleae; this cell character may affect the location of the cell engraftment.

The numbers of engrafted cells in WT mice and even in Cx30^−/−^ mice were relatively small; the numbers of engrafted cells were 0–10 per treated ear on E18.5. We think that there may be a few possible explanations for these small numbers of engrafted cells. The first possible explanation is that the cells injected into the otocysts might have leaked out after pulling out the capillaries. Indeed, we have experienced some leakage of dye from an injection hole after pulling out the glass capillaries. When we assessed cell existence in the otocysts immediate after the cell delivery, we found aggregated cells in one otocyst (Fig. 2B), while another embryo had a significantly smaller number of donor cells in the otocyst (Fig. 2A). Considering these findings, we assume that the cells that failed to be aggregated after otocystic injection might leak from the injection hole. Moreover, the volume that was injected into the otocyst is 0.1 to 0.2 μL per inner ear. Therefore, when we injected cells with a concentration of 1.0–5.0 × 10^6^/ml, the numbers of injected cells were theoretically 100–1000 per treated ear, at maximum. These numbers were probably too small to obtain a large number of engrafted cells, considering potential leakage. In addition, the small number of injected cells might be also disadvantageous for the induction of cell aggregation in the otocysts, which might cause more leakage of the injected cells, as mentioned above. Considering these potential explanations, if we can increase the cell concentration of the injected cells, we might also be able to increase the number of engrafted cells. The second potential explanation for low numbers of engrafted cells is that delivered cells might be eliminated after cell injection by some process other than cell leakage through the injection hole. Immune responses may be a factor that should be considered, even though the developing fetus is known to possess immunologic tolerance during the prenatal stage. There are indeed several reports describing successful hetero-transplantation of mouse-derived cells into chick otocysts^29–31^. The third possible process of cell elimination is related to the microenvironment of delivered cells. If the delivered cells failed to attach to or migrate into the recipient tissue, they presumably remained in a floating condition in the lumen of the embryonic inner ears. These floating cells probably do not obtain a sufficient supply of blood and growth factors for cell survival from the surrounding tissues and may die easily. Thus, modulation of adhesion molecules in the otocystic lining cells may improve survival and engraftment of the injected cells^32^. The forth potential explanation for the low numbers of cell engraftment may be related to the status of tissue damage in the inner ear of the recipient mice^28^. Cx30^−/−^ mouse embryos at E11.5 (which is prior to the initiation of Cx30 gene expression in the inner ear in WT mice) probably do not yet have any tissue damage in the inner ear. Considering this likelihood, cell injection to Cx30^−/−^ mice at E11.5 may be disadvantageous to successful cell engraftment. Therefore, transplanting cells at later embryonic stages or postnatal stages may help to increase the number of the engrafted cells.

Results from ABR testing in WT mice did not show any significant auditory threshold deteriorations on P30, suggesting that transuterine cell transplantation into the E11.5 otocysts per se does not cause harmful effects on auditory functions, at least technically. However, considering the finding that the numbers of engrafted cells in the WT mice were small in this study, we should further clarify how larger numbers of cells engrafted in the developing inner ears affect inner ear function.

The progenitors of OSCs, which were destined to differentiate into non-sensory cells, migrated to the non-sensory region both in wild type mice and in CX30^−/−^ mice in this study. In addition, some of the delivered progenitors of OSCs, which had the ability to develop into OSCs expressing CX30 in culture, differentiated to the cells expressing the CX30 protein. However, the number of the engrafted cells with CX30 protein expression was very low and one of the cells which located at the lesion where cells were not supposed to express CX30 proteins originally, had CX30 expression. In our *in vitro* culture study, we obtained nearly 80% of OSCs expressing CX30 protein from the progenitors of OSCs by treatments with NaHCO3, while only 12% (3 out of 25) of engrafted cells expressed CX30 *in vivo*. Considering this treatment process and the results of *in vitro* culture, the delivered progenitors of OSCs in this study probably did not obtain a sufficient microenvironment to develop into cells with CX30 protein expression after *in vivo* transplantation. If we were able to deliver progenitors of OSCs that had undergone NaHCO3 treatment *in vitro*, we may be able to obtain a higher engraftment rate of CX30-positive cells, and this process might simultaneously contribute the proper differentiation of the delivered cells according to the microenvironment of the engrafted location. In addition, the usage of mouse-derived cells as donor cells may improve the engraftment rate of delivered cells and the rate of proper CX30 protein expression.

For successful cell-based therapy for profound genetic hearing loss, the transplanted cells need to be engrafted into the areas where target cells exist in healthy individuals, and subsequently need to differentiate into the matured target cells in order to compensate for the function of the target cells. In this study, we explored the feasibility of embryonic cell transplantation therapy of CXs-related hearing loss, utilizing progenitors of OSCs that were derived from hiPSCs. We found that the delivered cells engrafted into non-sensory cell lesions in the cochleae both in WT mice and CX30^−/−^ mice. Moreover, we found significantly larger numbers of engrafted cells in CX30^−/−^ mice, compared with WT mice. The delivered progenitors of OSCs appeared to differentiate into cells with or without CX30 protein expression; however, the CX30 expression pattern appeared not to be related to the engrafted location. The higher engraftment in non-sensory cell lesions in CX30^−/−^ mice may work well for cell therapy of CXs-related genetic hearing loss. To achieve functional recovery in CX30^−/−^ mice, the next step would be to increase the number of engrafted cells with CX30 protein expression. This will presumably require the use of mouse-derived cells as donor cells and the precise determination of the appropriate differentiation stage of the delivered cells.

In summary, this is the first report demonstrating the successful engraftment of exogenous cells in the prenatal developing otocysts in mice. We show successful cell transplantation of progenitors of OSCs derived from hiPSCs into the mouse otocysts on E11.5. The delivered cells engrafted in the non-sensory region in the inner ear for up to 1 week after transplantation and expressed CX30 protein. While there remain obstacles to overcome regarding successful cell-based therapy, these findings provide some hope for the development of cell transplantation therapy for the treatment of profound genetic hearing loss caused by CXs deficiency.

## Material and Methods

### Ethics statement

All animal experiments were approved by the Committee on the Use and Care of Animals at Kumamoto University and were performed in accordance with accepted veterinary standards.

### Animals

Timed-pregnant wild type (WT) CD-1 mice were purchased from Japan SLC Inc. (Shizuoka, Japan), and CONNEXIN30 knockout (CX30^−/−^) mice were provided by Klaus Willecke (Teubner *et al*. 2013). The CX30^−/−^ on a C57BL/6 J background were backcrossed with WT mice on a CD-1 background until the 3rd generation. Third-generation CX30^−/−^ mice were maintained by crossbreeding CX30^−/−^ mice × CX30^−/−^ mice with genotyping, utilizing the Extract-N-Amp™ Tissue PCR Kits (Sigma, St. Louis, MO, USA). Noon on the day on which a vaginal plug was detected was designated as embryonic day 0.5 (E0.5) of development.

### Induction of progenitors of the outer sulcus cell-like cells *in vitro*

Human-derived iPS cells (hiPSCs), which were obtained from a healthy 16-year-old girl’s skin (WD39)^33^, were cultured under feeder-free conditions for 2 days at the pre-differentiation stage, and then those were cultured with Rho kinase inhibitor, FGF2, FGF3, FGF10, FGF19 and BMP4 for 9 days, which were differentiated into otic progenitor cells^15^. Consequently, those cells differentiated into otic progenitor cells (OPCs); the OPCs expressed PAX8, PAX2, SOX2, FOXG1, TBX1, OTX1, and GATA3, which was confirmed by immunocytochemistry and RT-PCR. The OPCs were then cultured with LW medium for 2 weeks and differentiated into cells containing progenitors of the OSCs (referred to as “progenitors of OSCs”)^15^. The progenitors of OSCs were maintained in 6-well dishes coated with Poly O (Sigma) and Fibronectin (Sigma) in the medium including DMEM (Sigma), 8% FBS (Biological Industries, Haemek, Israel), bFGF (Peprotech, NJ, USA), 1 M HEPES (Sigma), and 0.1% Ampicillin (Thermo Fisher Scientific, MA, USA), which was changed every 3 days before confluency. The progenitors of OSCs were collected in microtubes, which were filled with DMEM; the final concentration of the progenitors of OSCs for transplantation was 1.0~5.0 × 10^6^/ml. We utilized theses progenitors of OSCs (Supplementary Figure 1) for cell transplantation into the otocysts. The size of the progenitors of OSCs was measured with ImageJ software (NIH, Bethesda, MD, USA; Supplementary Figure 1C).

### Induction of the outer sulcus cell-like cells *in vitro*

To confirm the developmental abilities of the progenitors of OSCs to mature into OSCs, the induction to OSCs was performed *in vitro*, following the method described in a previous report^15^. Briefly, the medium of the progenitors of OSCs, which consisted of DMEM (Nacalai Tesque, 08459-64) containing 10% FBS, 0.75% NaHCO_3_, and 50 U/mL penicillin/streptomycin, was changed every 2 days for 1 week. Subsequently, the concentration of NaHCO_3_ in the medium was changed to 0.375%, and the cells were maintained for another week (Fig. 1).

### Cell Counts

OPCs, progenitors of OSCs, and OSCs were immunostained for otic progenitor markers (PAX2, PAX8, and SOX2) and CX30. To quantify the ratio of cells expressing these markers, we counted the number of cells which had nuclei positive for all three progenitor markers and cells which were positive for CX30 in three or four randomly selected areas and normalized to the total number of cells counted using Hoechst33258. More than 50 cells were counted in each area. Averages of the ratio are shown in the graph.

### Cell transplantation into the E11.5 otocysts

Pregnant mice were anesthetized, on E11.5, with intraperitoneal administration of 10 mg/kg of xylazine (Bayer, KS, USA) and 90 mg/kg of ketamine-HCl (Daiichisankyo, Tokyo, Japan) in saline. Details of the intrauterine injection into mice otocysts were described previously^2,34,35^. Progenitors of OSCs, which were used for transplantation, were dissociated from 6-well dishes into single cells using a 0.25% trypsin/0.02% EDTA solution and pipetting procedure. Briefly, each uterus was illuminated with a fiber-optic beam to identify the otocyst (Fig. 1 and Supplementary Figure 2B) after laparotomy, and subsequently, the cell admixture with 1% fast green (Sigma, St. Louis, Missouri, USA) as a tracking dye was injected by oral pressure into the right-side otocysts in one or two embryos per dam through the uterine wall using heat-pulled 20- to 30-μm glass microcapillaries (Fig. 1 and Supplementary Figure 1B). After the injections, the embryos were gently returned into the abdominal cavity and the abdominal skin was sutured with 30 mg/kg of chloramphenicol. For postnatal assessments, the treated embryos on E18.5 were passed to surrogates for further fostering.

### Determination of tip size of glass microcapillaries

To determine the appropriate tip size of the glass microcapillary for otocystic injection, we performed transuterine otocystic injection of 1% fast green in saline using three kinds of glass microcapillaries, whose tip sizes were 40, 30, or 20 μm of internal diameter (I.D.). Immediately (within 1 min) after the cell injections, the dams were euthanized by intraperitoneal administration of overdose of 10 mg/kg of xylazine (Bayer, KS, USA) and 90 mg/kg of ketamine-HCl (Daiichisankyo, Tokyo, Japan) in saline. To assess the density of fast green in the otocysts, the embryos were removed, and images of the body surface of the embryos were captured and stored on a personal computer. We firstly measured the diameter of single cells of progenitor of OSCs and found it to range from 10 to 25 μm (Fig. 2C). When we used 40-μm glass microcapillaries for otocystic injection, we could not detect any dye stain in the otocysts (Supplementary Figure 1E), suggesting complete leakage of the injected cells from an injection hole. When we used 30-μm (Supplementary Figure 1D) or 20-μm glass microcapillaries (Supplementary Figure 1A,C), we found significant dye staining in the otocysts. We therefore decided to use 20~30-μm glass microcapillaries for the following cell injection experiments, to decrease the leakage of cells from an injection hole after pulling out the capillaries, while avoiding clogging of the glass microcapillaries with cells.

### Cryosectioning

To assess the delivered cells in the otocysts using cryosectioning, the treated embryos or postnatal mice were euthanized 1 h after cell injections, on E15.5, E18.5, and P30 in the WT-treated group, and on E18.5 in the CX30^−/−^ treated group. Briefly, the samples were fixed in 4% paraformaldehyde for 12 h at 4 °C. Regarding the inner ears of postnatal mice, the samples were decalcified for 2 days at room temperature by EDTA, after the fixation, subsequently embedded in OCT medium (Sakura Finetek Japan, Tokyo, Japan), and then serially sectioned to a thickness of 12 μm.

### Immunostaining

For the *in vitro* study, the cells were fixed with 4% paraformaldehyde, then boiled in 0.1 mM citrate buffer (pH 6.0) for 1 h and blocked with PBS containing 10% normal goat or donkey serum for 1 h at room temperature. The cells were incubated with primary anti-CX30 (rb, 1:100, Sigma, HPA014846) or anti-PENDRIN (gt, 1:100, Santa Cruz, SC23779) antibodies at 4 °C overnight. After three washes with PBS, the cells were incubated with Alexa 488-, Alexa 555-, or Alexa 647-conjugated secondary antibodies (Life Technologies) for 1 h at room temperature. The nuclei were stained with 10 mg/mL Hoechst 33258 (Sigma). After washing with PBS, the cells were examined using a confocal laser scanning microscope (LSM700; Carl Zeiss) or a BZ-9000 fluorescence microscope (Keyence). Images were captured and stored on a personal computer.

For the *in vivo* study, cryosectioned slices were incubated with Hoechst 33258 dye (Molecular Probes, Eugene, OR, USA) for nuclei staining and/or Texas Red-phalloidin (Thermo Fisher Scientific, Waltham, MA, USA), after fixing with 4% paraformaldehyde and blocking with 0.1% Triton-X (Triton X-100 in PBS; IBI Scientific, Peosta, IA, USA). During each process, the tissues were washed two times for 5 min with PBS. The slices were immunoreacted with human nucleic-specific antigen STEM101 (ms, 1:50; Takara Bio Inc., Shiga, Japan) as a primary antibody and Alexa 488-, 532-, or 594-conjugated secondary antibodies (1:2000; Thermo Fisher Scientific) to identify the human-derived cells. During STEM101 staining, a M.O.M. Immunodetection kit (Vector Laboratories, Burlingame, Ca, USA) was utilized to decrease the non-desirable background staining. We defined the engrafted cells those which were double-positive for STEM101 and Hoechst staining in the nuclei. To determine CX30 protein expression in donor cells, serially sectioned slices, which were obtained from three E18.5 embryos in the WT-treated group and two E18.5 embryos in the CX30-treated group, were immunoreacted with a polyclonal anti-CX30 primary antibody (rb, 1:200; Thermo Fisher Scientific) and an Alexa 532-conjugated secondary antibody (1:2000; Thermo Fisher Scientific). The samples were examined under a BZ-9000 fluorescence microscope (Keyence, Osaka, Japan). Images were captured and stored on a personal computer.

### Auditory brainstem responses (ABRs)

Auditory thresholds were measured using the ABR System 3 (Tucker-Davis Technologies, FL, USA). The animals were anesthetized by the intraperitoneal administration of xylazine and ketamine-HCl in saline. Electrodes were placed beneath the pinna of the treated ear and at the vertex just below the surface of the skin, and the ground electrode was then placed under the contralateral ear. An average of 512 sweeps was calculated for 4, 8, 12, and 20 kHz. The stimulus levels near the threshold were varied in 5-dB steps, and the threshold was defined as the lowest level at which waves in the ABR could be clearly detected by visual inspection. Each *n* = 5.

### Statistical analyses

Data were analyzed statistically using the Mann-Whitney U test. Data are presented in the text and figures as means ± standard error (SE). The statistical significance level was set at p < 0.05.

## Electronic supplementary material

Supplementary information
