# Supplementary material for: Engraftment of Human Pluripotent Stem Cell-derived Progenitors in the Inner Ear of Prenatal Mice
Source: Sci Rep. 2018 Jan 31;8:1941. doi: 10.1038/s41598-018-20277-5 (PMC5792596; doi:10.1038/s41598-018-20277-5)
Supplement: Supplementary file 1 — Supplementary information [file 41598_2018_20277_MOESM1_ESM.pdf]

Successful Engraftment of Human Pluripotent Stem Cell-derived Progenitors in the Inner Ear of Prenatal Mice

Hiroki Takeda[1], Makoto Hosoya[2], Masato Fujioka[2], Chika Saegusa[2], Tsubasa Saeki[2], Toru Miwa[1,4], Hideyuki Okano[3] and Ryosei Minoda[1,4]*.

[1]Departments of Otolaryngology-Head and Neck Surgery Kumamoto University, Graduate School of Medicine, 1-1-1 Honjo, Chuoku, Kumamoto city, Japan

[2]Departments of Otolaryngology-Head and Neck Surgery, Keio University School of Medicine, 35 Shinanomachi, Shinjuku-ku, Tokyo, Japan

[3]Department of Physiology, School of Medicine, Keio University, 35 Shinanomachi, Shinjuku, Tokyo, Japan.

[4]Departments of Otolaryngology-Head and Neck Surgery, Middle Ear and Inner Ear Surgical Center, JCHO Kumamoto General Hospital, 10-10 Tori Machi, Yatsushiro, Kumamoto, 866-8660, Japan

Corresponding author: Ryosei Minoda, Ph.D., M.D.
Departments of Otolaryngology-Head and Neck Surgery, Middle Ear and Inner Ear Surgical Center, JCHO Kumamoto General Hospital
Correspondence should be addressed to R.M. (ryminoda@gmail.com).
Corresponding author's address: 10-10, Tori Machi, Yatsushiro, Kumamoto, 866-8660, Japan

Supplementary figures and figure legends

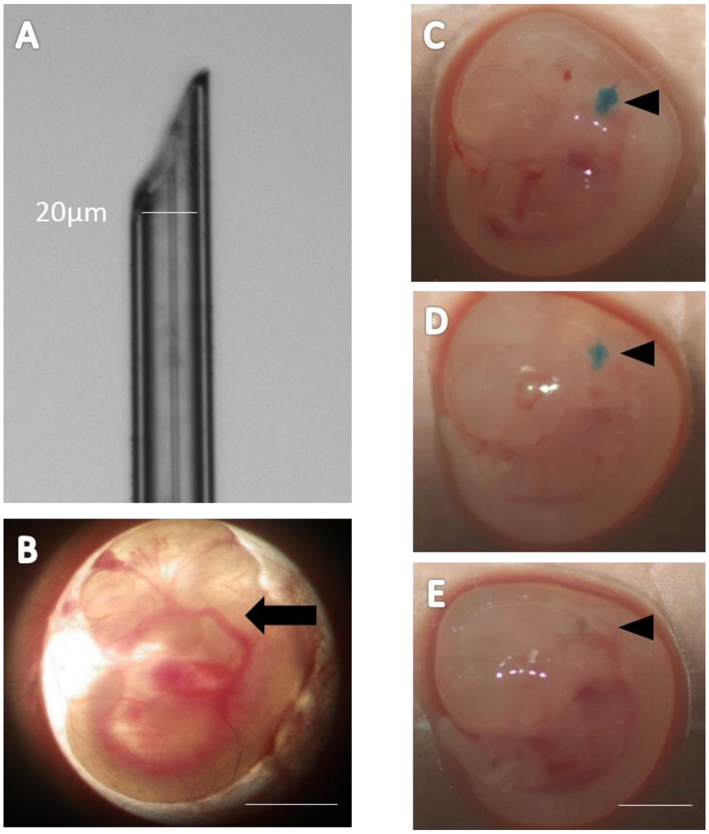

Supplementary Figure 1.

(A) The image of an ideally shaped glass micropipette for cell transplantation. The diameter of the tip is about 20 μm. **(B)** Picture of a mouse embryo on E11.5 in the visceral yolk sac without the uterus. An arrow indicates the location of the otocyst. The bar indicates 1000 μm. **(C-E)** Picture of a mouse embryo after fast green injection on E11.5 without the uterus, the visceral yolk sac, and the amnion. Arrowheads indicate the otocyst filled with fast green using a 20-μm glass micro pipette (C) and a 30-μm glass micro pipette (D), and the otocyst not filled with fast green using a 40-μm glass micro pipette (E). The bar indicates 1000 μm.

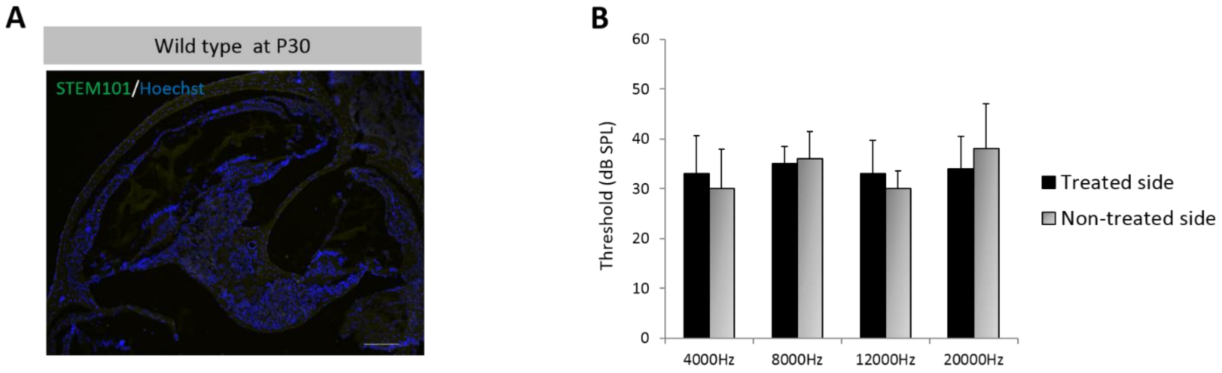

Supplementary Figure 2.

(A) The image shows the section of the treated cochlea on P30 in the WT-treated group. No transplanted cells were detectable. Green: STEM101; Blue: Hoechst. Scale bar indicates 100 µm. **(B)** ABR testing results on P30 after transplantation in the WT-treated group. No significant difference was found between the treated and the non-treated groups at 4, 8, 12, and 20 kHz. Each *n*=5.
